# Histological Evaluation of Placentas in Idiopathic Intrauterine Growth Restriction

**DOI:** 10.7759/cureus.72789

**Published:** 2024-10-31

**Authors:** Saadi S Barwari

**Affiliations:** 1 Anatomy and Histology, College of Medicine, University of Duhok, Duhok, IRQ; 2 Basic Medical Sciences, College of Medicine, University of Zakho, Zakho, IRQ

**Keywords:** embryology, fetus, histopathology, idiopathic iugr, placenta

## Abstract

Background

Idiopathic intrauterine growth restriction (IUGR) is a condition in which there is no discernible cause, such as problems with the mother's health, and the fetus does not grow to the expected size for its gestational age. In cases of IUGR, the placental trophoblast exhibits reduced invasiveness, leading to a less extensive invasion of uterine spiral arteries and increased resistance in the uteroplacental circulation. The consequences of these early histopathological alterations are long-lasting, resulting in compromised blood flow to the placenta and diminished transport of nutrients and oxygen from the mother to the fetus. The placentas of neonates with idiopathic IUGR may provide crucial insights into the underlying causes of this growth restriction. The present study was designed to evaluate various microscopical changes in placentas of idiopathic intrauterine growth-restricted cases, qualitatively and quantitatively.

Methods

After getting the ethics committee's approval, the placental samples were collected from Duhok Obstetrics and Gynecology Hospital, Duhok, Iraq. A total of 55 placentas were gathered from women aged 20-40 years who had full-term (37-40 weeks) singleton pregnancies. Control cases were selected randomly, while idiopathic IUGR cases were selected purposively. Out of 55, 35 placentas were taken from idiopathic IUGR (birth weight less than 2500 g), and 20 placentas were taken from normal pregnancies (control group) with no history of confounding maternal and fetal factors. The maternal and neonatal data (age, weight of neonate, gestational period, and gender of neonate) were recorded. Immediately after delivery of the baby, the placenta was taken, washed with tap water, and dried. Then central and peripheral biopsies were taken for qualitative and quantitative histopathological examination, which includes the following: the number of terminal villi, stromal fibrosis, fibrinoid necrosis, syncytial knots, hyalinization, chorangiosis, congestion, and calcification. Statistical analyses were done using Student's t-tests and chi-square tests.

Results

In the idiopathic IUGR group, the qualitative features of the microscopic study corresponded with quantitative measurements. There was a significant decrease in the mean number of terminal villi (p=0.02), a highly significant increase in syncytial knots (p=0.001), a highly significant increase in fibrinoid necrosis (p=0.003), a highly significant increase in the mean number of stromal fibrosis (p=0.001), and a significant decrease in hyalinization (p=0.05). There was an insignificant increase in the calcification, medial coat proliferation of medium-sized blood vessels, chorangiosis, congestion, and fatty degeneration.

Conclusions

The reduction of terminal villi, accompanied by an increase in syncytial knots, fibrinoid necrosis, and stromal fibrosis, may have reduced the surface area for fetomaternal exchange. This led to chronic placental insufficiency. As a result, fetal growth and development are restricted. Therefore, IUGR infants may need more scientific and clinical attention.

## Introduction

The placenta is a link between the mother and her fetus, so it consists of the following two parts: the maternal part, "decidua basalis," and the fetal part, "chorion frondosum." The maternal component of the placenta consists of the endometrium, which is called decidua after implantation. On the other hand, the fetal component of the placenta arises from the surrounding trophoblast, which transforms into chorion after the development of the extraembryonic mesoderm. Most of the chorionic villi disappear from the chorion except for the part called the chorion frondosum that is attached to the decidua basalis. The chorion frondosum joins with the maternal decidua basalis by the cytotrophoblastic shell and anchoring villi to form the placenta, also called choriodecidual membrane [1].

Intrauterine growth restriction (IUGR) is the term used to describe a fetus that fails to reach its full growth potential compared to that expected for its gestational age. Both the American Congress of Obstetricians and Gynecologists (ACOG) and the Royal College of Obstetricians and Gynecologists (RCOG) define intrauterine growth restriction as an estimated fetal weight less than the 10th percentile [2].

Histopathology studies have shown that idiopathic IUGR-affected placentas are typically smaller than gestation-matched controls, suggesting that atypical placental development may be an important contributing factor to IUGR [3]. Familial factors and a possible genetic role have been proposed by some epidemiological studies in human idiopathic IUGR [4].

In IUGR, the placental trophoblast is less invasive, leading to relatively low invasion of uterine spiral arteries and increased uteroplacental vascular resistance. These early events result in compromised uteroplacental perfusion and reduced maternal-fetal transport of nutrients and oxygen [5,6]. Placentas in IUGR pregnancies are characterized by several histopathological phenotypes, such as chronic villitis, avascular villi, villous infarction, distal villous hypoplasia, perivillous fibrinoid deposition, cytotrophoblast hyperplasia, syncytial knots, chorangiosis, and basement membrane thickening [6,7]. A study revealed a significant increase in syncytial knots and capillaries in the terminal villi of IUGR placentas compared to the control group [8]. Another study observed a significant correlation between IUGR and placental arterial narrowing, degeneration, decreased number of arteries in stem villi, decreased number of terminal villous capillaries, and villitis [9]. In fact, intrauterine growth restriction is associated with fetal hypoxia [9]. Placental factors and hypoxemia contribute to IUGR and fetal death.

In general, few studies have investigated the involvement of the placenta in IUGR. There are even fewer studies on idiopathic IUGR. Furthermore, no previous research has investigated the specific histological changes associated with IUGR in our region. Therefore, the present study was designed to evaluate the various microscopic changes “qualitatively and quantitatively” in the idiopathic IUGR placenta.

## Materials and methods

This cross-sectional study was conducted in the Department of Anatomy and Histology, College of Medicine, University of Duhok, and Duhok Maternity Hospital, Duhok, Kurdistan region, Iraq. The study was approved by the Institutional Review Board of Duhok Directorate of General Health and Directorate of Planning, Scientific Research Division, and all placental samples were obtained after the patient's agreement.

Sample collection

All placental samples were collected at Duhok Maternity Hospital, Duhok City. The tissue processing was done in the Central Laboratory, Duhok City. The period of collection of samples was from December 1, 2015, till June 1, 2016. During this period, a total of 55 placentas were collected from women aged 20-40 years for both the control and idiopathic IUGR groups of full-term (37-41 weeks gestational age) singleton pregnancies. Control cases were selected randomly, while idiopathic IUGR cases were selected purposively. Of the 55 placentas, 35 were obtained from idiopathic IUGR (birth weight less than 2500 g) pregnancies, and 20 placentas were taken from the control group, which were obtained from normal-term pregnancies without any complications.

Demographic and clinical data were extracted from maternal and neonatal medical records. All patients were delivered by cesarean section (CS) (emergency and elective type of CS), and all infants were live-born and without any evidence of congenital infection or malformation.

Inclusion and exclusion criteria

Placental samples from women aged 20-40 years with full-term singleton pregnancies were included in both the control and idiopathic IUGR groups. The placental samples from the following cases were excluded: multiple pregnancies, preterm pregnancies, placental tumors, trauma, and any cases complicated by chronic diseases such as hypertension, diabetes, cardiovascular diseases, and renal disease. Additionally, pregnant women who smoked and/or consumed alcohol were excluded. Women with antenatal hemorrhage (including abruptio placentae, vasa previa, and placenta previa) were also excluded from the study. Finally, any patient who disagreed with the use of her placenta in the study was excluded.

Microscopic preparation and examination of placental sections

The placenta was cut along its maximum diameter into two parts. Each part was further cut into three pieces (3 mm thick slices). Specimens, 2-3 pieces, were randomly selected (one from the periphery, the second from the center, and the third one if there is any abnormal lesion like hemorrhage or infarction) with full thickness, including both fetal and maternal sides. All specimens were preserved in 10% neutral buffered formalin (Hyde, UK: Atom Scientific). Containers were then labeled with code numbers and left for 24 hours at room temperature. Then, the 3 mm-thick slices were transferred into labeled plastic tissue cassettes, and they were put into the automated processor (LEICA ASP 300S; Karlsruhe, Germany: Leica Biosystems). Tissue processing, preparation of 5 µm thick sections, staining with hematoxylin and eosin, and mounting were achieved according to Suvarna et al. [10]. Stained sections were examined by the light microscope (Nikon Y-THS; Minato, Japan: Nikon) at magnifications ×40 and ×100. Both qualitative and quantitative changes were recorded.

Qualitative and quantitative examination

First, the placental slide sections of the control group were carefully examined. The examination focused on all histological components, including maternal and fetal parts, the amnion, chorionic plate, chorionic villi (large stem villi to small terminal villi) and their stroma, fetal blood vessels, syncytial knots, fibrinoid necrosis, fibrosis, villous edema, infarction, calcification, hyalinization, and any other changes on tissues. All the abovementioned structures were measured and compared with cases of idiopathic IUGR placentas.

From each section, 10 fields were taken [11]. Each section starts from the maternal side to the fetal side randomly to calculate the number of terminal villi, syncytial knots, fibrinoid necrosis, stromal fibrosis, hyalinization, congestion, edema of the villous, infarction, calcification, chorangiosis, and medial coat proliferation of medium-sized blood vessels. The appropriate stained sections were photographed by using a digital camera (Nikon Digital Sight DS-SM; Minato, Japan: Nikon).

Statistical analysis

The data were analyzed using SPSS version 20 (Armonk, NY: IBM Corp.). A two-tailed Student's t-test was used for independent samples to compare the means of the following numerical data: the number of fibrinoid necrosis, medial coat proliferation of medium-sized blood vessels and terminal villi, stromal fibrosis, hyalinization, syncytial knot formation, and calcification. The chi-square (χ²) test was used to measure the following categorical data and to differentiate between them: qualitative variables such as infarction, calcification, fibrinoid changes, congestion, and chorangiosis. Fisher’s exact test was applied when the chi-square test was not applicable (i.e., when more than 20% of cells had an expected or observed count of less than five). All analyses were deemed statistically significant when the p-value was ≤0.05.

## Results

Qualitative observations

**Figure 1:**
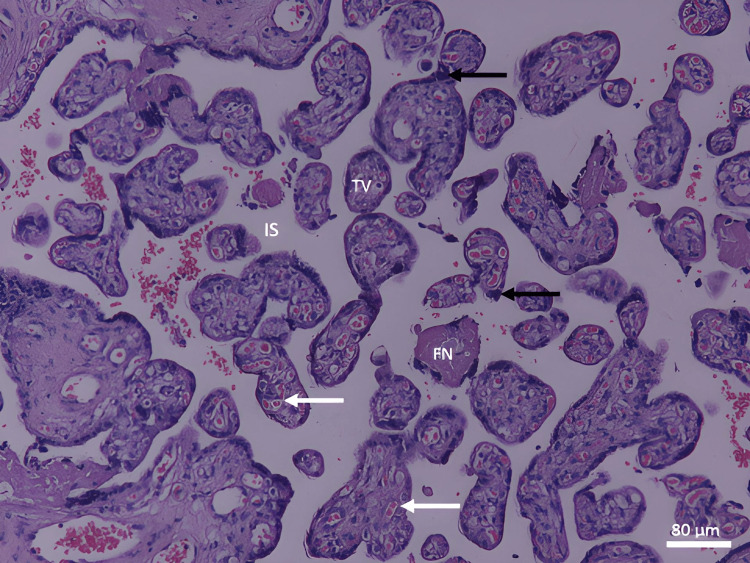
A photomicrograph of a normal placenta shows TV with a core of connective tissue rich in fetal capillaries (white arrows) and separated from each other by IS. Depicted are the syncytial knots (black arrows) and the FN (H&E, ×100). TV: terminal villi; IS: intervillous spaces; FN: fibrinoid necrosis

**Figure 2:**
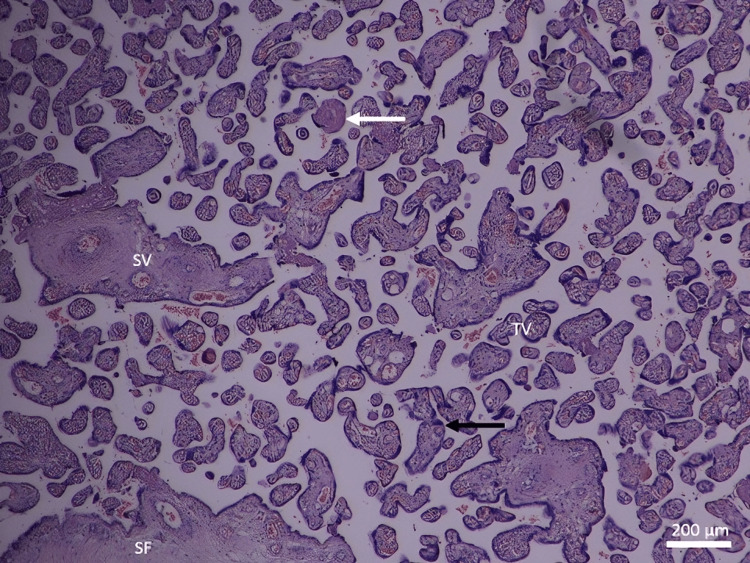
A photomicrograph of normal placenta shows TV and large SV with SF. Depicted are the syncytial knots (black arrow) and fibrinoid necrosis (white arrow) (H&E, ×40). TV: terminal villi; SV: stem villi; SF: stromal fibrosis

In the control group, the full-term placenta appeared to have terminal villi as a small, rounded, or oval structure with a connective tissue core surrounded by a continuous layer of syncytiotrophoblast and underneath an area of a few cytotrophoblast cell layers (Figure 1). There were many fetal capillaries in the terminal villi, and there were intervillous spaces between them that were filled with maternal blood. Syncytial knots were occasionally observed attached to terminal villi, free in the intervillous spaces, and between villi. There was a possible amount of fibrinoid deposition that was present between the villi, covering the villi (perivillous fibrinoid), in the core of the villi (intravillous fibrinoid), at the junctional zone, and in the chorionic plate (Figures 1, 2).

The stem chorionic villi were much larger than the terminal villi. They were characterized by the presence of small arteries and veins with a well-defined muscular wall, few arterioles and venules, and fewer capillaries. In addition, there was a large amount of connective tissue stroma with fibroblasts and a fewer number of trophoblast cells on their periphery. The microscopic analysis revealed a significant decrease in the IUGR group's mean number of terminal villi. Some areas of stromal fibrosis were also present in the villous core and around blood vessels (Figures 1, 2). Obliterative changes in the small capillaries of the villi were present. Some chorangiosis, congestion, and calcification showed up in a few cases.

**Figure 3:**
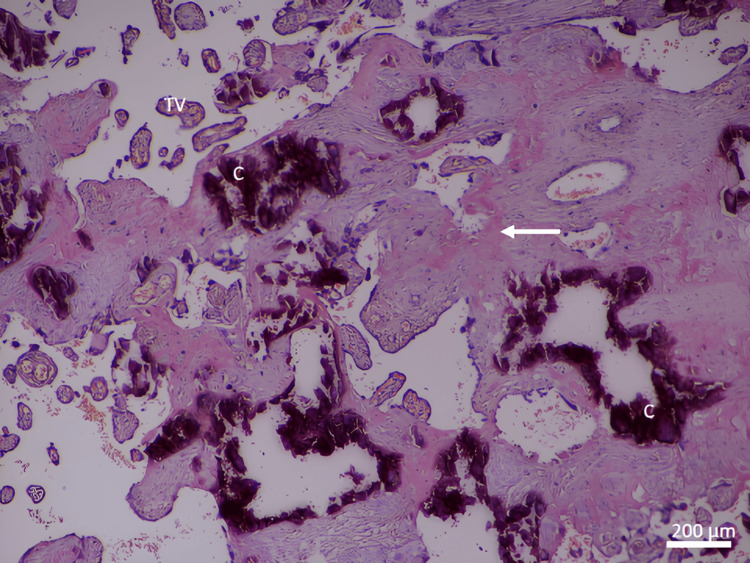
A photomicrograph of an idiopathic IUGR placenta shows an increase in calcification and fibrinoid necrosis (arrow), and a decrease in the number of TV (H&E, ×40). C indicates calcification. TV: terminal villi; IUGR: intrauterine growth restriction

**Figure 4:**
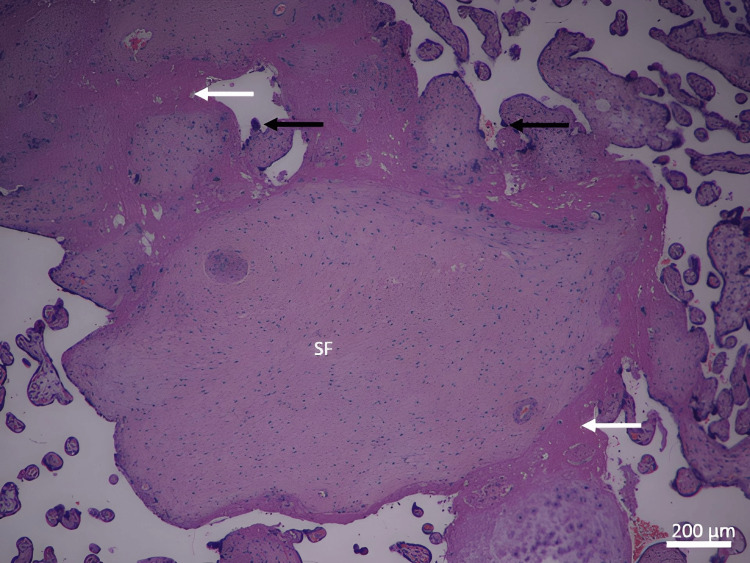
A photomicrograph of an idiopathic IUGR placenta shows an increase in SF and fibrinoid necrosis (white arrows). Depicted is the high number of syncytial knots (black arrows) (H&E, ×40). SF: stromal fibrosis; IUGR: intrauterine growth restriction

**Figure 5:**
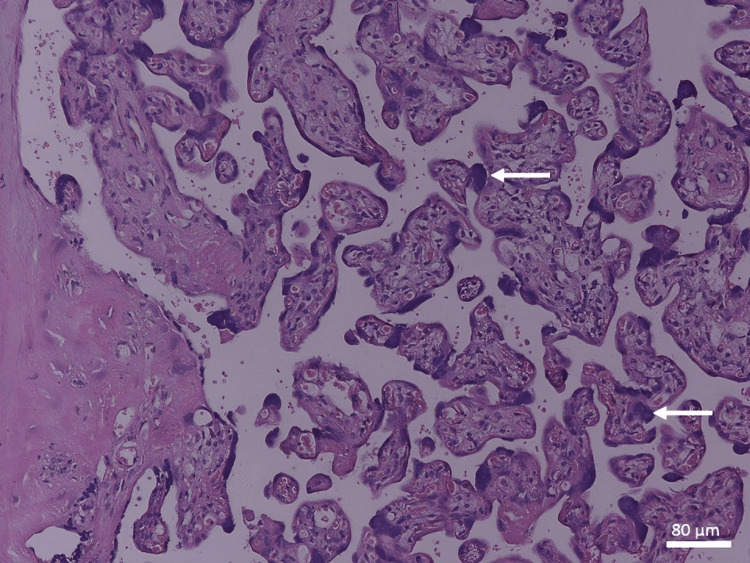
A photomicrograph of an idiopathic IUGR placenta shows increased numbers of syncytial knots (arrows) (H&E, ×100). IUGR: intrauterine growth restriction

In contrast, the specific histological changes found in idiopathic IUGR placentas were as follows: terminal villi decreased in number and diameter (Figure 3). There was an increased incidence of fibrinoid necrosis in the central and peripheral areas of the placenta (Figures 3, 4). The nuclei of trophoblasts showed some changes, with a tendency toward the formation of clusters, especially when the syncytial layer progresses, buds (knots) protruding into the intervillous spaces, and cytotrophoblastic cellular proliferation (increased syncytial knot formation) (Figure 5).

**Figure 6:**
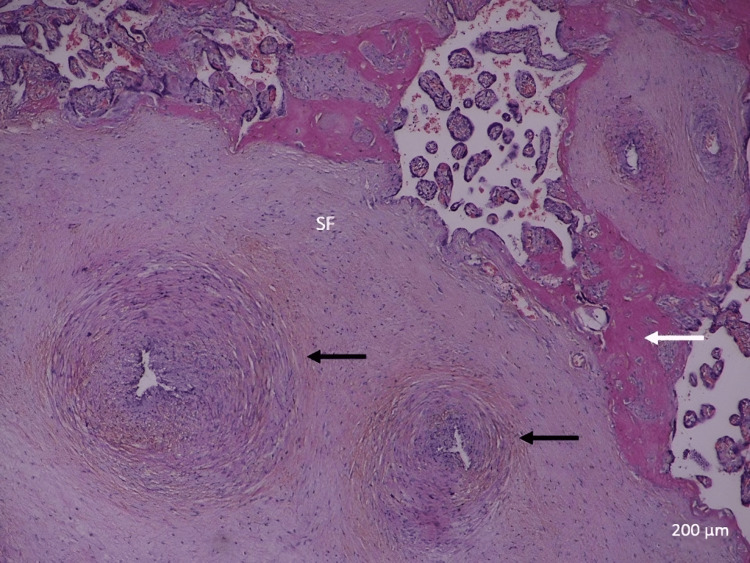
A photomicrograph of an idiopathic IUGR placenta. Depicted is the medial coat proliferation of medium-sized blood vessels (black arrows) which led to the narrowing of their lumens, and SF and fibrinoid necrosis (white arrow) were increased (H&E, ×40). IUGR: intrauterine growth restriction; SF: stromal fibrosis

**Figure 7:**
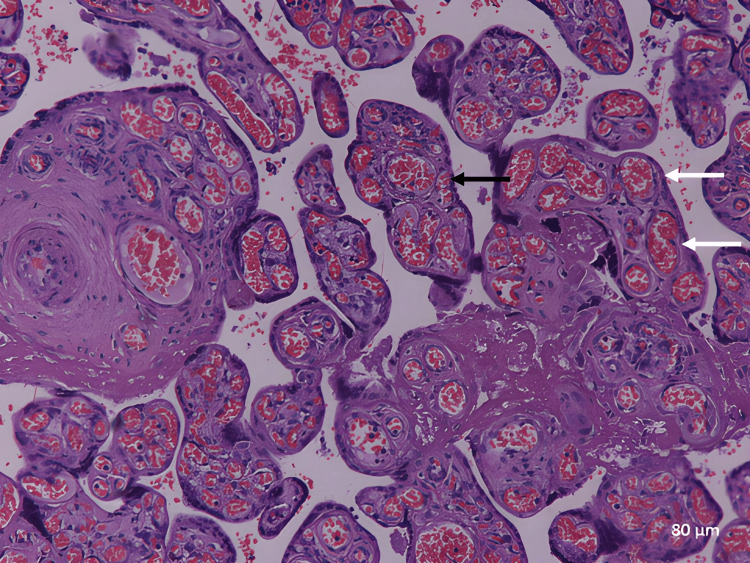
A photomicrograph of an idiopathic IUGR placenta shows congested and dilated villous capillaries with fetal blood (white arrows) and chorangiosis (black arrow) (H&E, ×100). IUGR: intrauterine growth restriction

Medial coat proliferation of medium-sized blood vessels increased (Figure 6). There was an increase in fetal blood capillary numbers (chorangiosis) and an increase in congestion of fetal blood capillaries with fetal blood (Figure 7).

**Figure 8:**
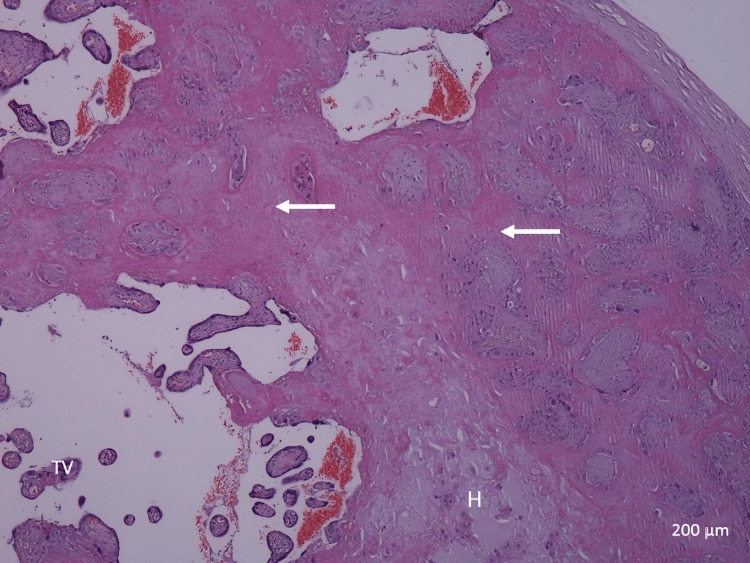
A photomicrograph of an idiopathic IUGR placenta shows the presence of hyalinization (H), an increase in fibrinoid necrosis (white arrows), and a decreased number of (TV) (H&E, ×40). TV: terminal villi; IUGR: intrauterine growth restriction

**Figure 9:**
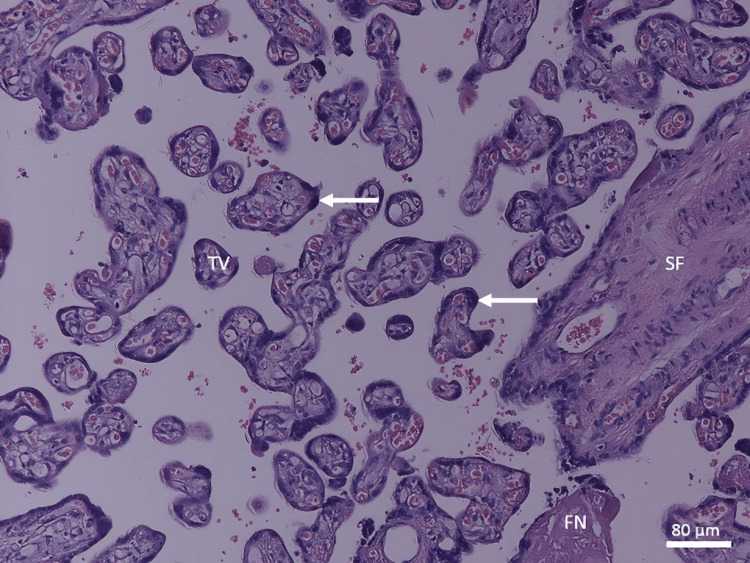
A photomicrograph of an idiopathic IUGR placenta shows a decreased number of TV, an increased number of syncytial knots (arrows), and the presence of FN and SF (H&E, ×100). IUGR: intrauterine growth restriction; TV: terminal villi; FN: fibrinoid necrosis; SF: stromal fibrosis

**Figure 10:**
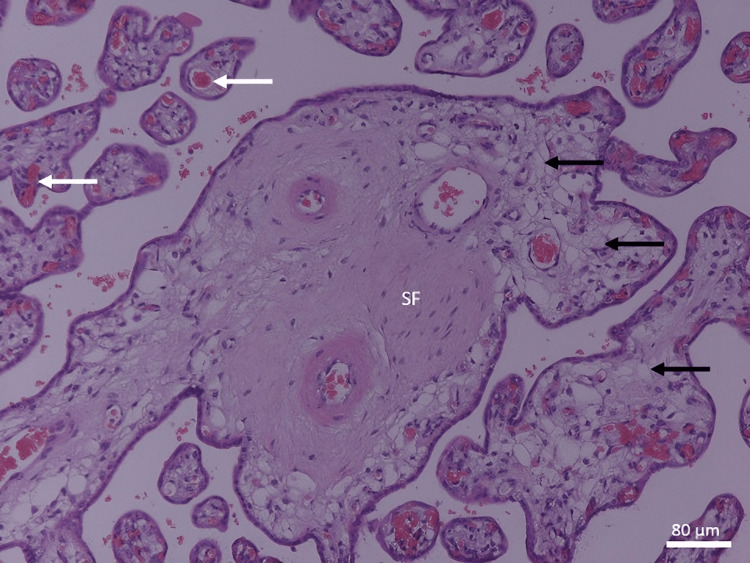
A photomicrograph of an idiopathic IUGR placenta shows increased fatty degeneration in the core of villi (black arrows), SF, and congestion of villous capillaries with fetal blood (white arrows) (H&E, ×100). IUGR: intrauterine growth restriction; SF: stromal fibrosis

Stem villi appeared to be increased in thickness with condensed villous connective tissue core. Hyalinization and stromal fibrosis were present extensively in the stroma of villous connective tissue (Figure 8). Calcification occurred in more cases of idiopathic IUGR in the core of the villi and in the decidua basalis, in contrast to the normal cases, where it was less present (Figure 3). There was degeneration of the endothelial wall of stem villi blood vessels with all stages of endothelial wall degeneration and fibrosis. There was an increased incidence of fibrinoid necrosis in perivillous and intravillous areas (Figure 9). There was an increased incidence of fatty degeneration in the core of villi (Figure 10).

Quantitative observations

There were several histological changes observed, including the number of terminal villi, syncytial knot formation, stromal fibrosis, fibrinogen necrosis, hyalinization, congestion, chorangiosis, fatty degeneration, medial coat proliferation of medium-sized blood vessels (MCP), and calcification. The critical value for significance was p≤0.05.

The microscopic analysis revealed a significant decrease in the mean number of terminal villi in the IUGR placentas when compared with the control placentas, p=0.02. Regarding syncytial knot formations, the mean number was significantly higher in the IUGR group than in the control group, p=0.001. There was a highly significant increase in the mean number of fibrinoid necrosis (p=0.003) in the idiopathic IUGR group when compared with the control group. The mean number of hyalinizations was significantly lower in the idiopathic IUGR group than in the control group, p=0.05. The mean number of calcification was insignificantly higher in the idiopathic IUGR group than in the control group, p=0.34. The mean number of stromal fibrosis was significantly higher in the idiopathic IUGR group than in the control group, p=0.001. The mean number of MCP was insignificantly higher in the idiopathic IUGR group than in the control group, p=0.57 (Table 1).

**Table 1:** Histological changes of placentas in control and idiopathic IUGR groups. P≤0.05 is considered statistically significant. IUGR: intrauterine growth restriction

Parameters	Control (mean±SD)	IUGR (mean±SD)	p-Value
Terminal vlli	13.16±2.98	10.81±2.30	0.02
Syncytial knots	9.86±2.38	12.47±1.63	0.001
Fibrinoid necrosis	8.23±3.91	10.73±2.15	0.003
Hyalinization	3.2±1.96	2.32±1.35	0.05
Calcification	1.45±2.41	2.21±3.09	0.34
Stromal fibrosis	3.58±1.29	5.16±1.57	0.001
Medial coat proliferation of medium-sized blood vessels	6.8±1.58	7.18±2.81	0.57

Of the total 55 cases studied microscopically, chorangiosis was found in 15 cases of idiopathic IUGR placentas and in nine cases of the control group, p=0.87, which is not statistically significant; congestion was found in 20 cases of idiopathic IUGR placentas and in seven cases of control groups, p=0.11, which is not statistically significant; and fatty degeneration has been found in 14 cases of idiopathic IUGR placentas and in four cases of control groups, p=0.12, which is not statistically significant (Table 2).

**Table 2:** Distribution of chorangiosis, congestion, and fatty degeneration in placentas of control and idiopathic IUGR groups. P≤0.05 is considered statistically significant. IUGR: Intrauterine growth restriction

Parameters	Control	IUGR	p-Value
Chorangiosis	9	15	0.87
Congestion	7	20	0.11
Fatty degeneration	4	14	0.12

The present study showed that there were no statistically significant differences in the mean occurrence of each studied histological change in the placentas of the four age groups of the idiopathic IUGR and control groups (Table 3).

**Table 3:** Microscopical changes of placenta in idiopathic IUGR group in relation to different age groups of mother. P≤0.05 is considered statistically significant. IUGR: intrauterine growth restriction; LSD: least significant difference test; NS: non-significant

Microscopical changes	Age group (year)	No.	Mean	SD	p-Value	Significance (LSD test)
Number of terminal villi	20-25	8	9.60	0.97	0.15	NS
	26-30	12	11.78	2.38		
	31-35	9	10.24	2.69		
	36-40	6	11.33	2.25		
	Total	35	10.81	2.30		
Syncytial knots	20-25	8	11.76	1.50	0.43	NS
	26-30	12	12.51	1.57		
	31-35	9	13.08	1.95		
	36-40	6	12.40	1.35		
	Total	35	12.47	1.63		
Fibrinoid necrosis	20-25	8	10.92	3.02	0.9	NS
	26-30	12	10.93	2.10		
	31-35	9	10.25	1.97		
	36-40	6	10.80	1.48		
	Total	35	10.73	2.15		
Stromal fibrosis	20-25	8	5.05	1.67	0.28	NS
	26-30	12	5.85	1.81		
	31-35	9	4.67	1.44		
	36-40	6	4.66	0.66		
	Total	35	5.16	1.57		
Medial coat proliferation of medium sized blood vessels (MCP)	20-25	8	5.63	1.68	0.15	NS
	26-30	12	7.87	2.59		
	31-35	9	6.64	3.51		
	36-40	6	8.68	2.65		
	Total	35	7.18	2.81		
Hyalinization	20-25	8	2.92	1.52	0.41	NS
	26-30	12	2.20	1.38		
	31-35	9	2.35	1.35		
	36-40	6	1.70	0.99		
	Total	35	2.32	1.35		

## Discussion

Placental evaluation is critical for understanding IUGR's pathophysiology. The underlying causes and risk of recurrence can be comprehended through comprehensive histologic evaluation, along with clinical-pathologic correlation [12]. Most cases of IUGR are associated with placental insufficiency [13]. The current research and recent studies give solid evidence of typical histological structural abnormalities in placentas affected by IUGR, despite other studies reporting no credible histologic abnormalities in the placentas of IUGR pregnancies [6,13].

Microscopically, examination of the placenta revealed significant differences in some histopathological parameters and non-significant differences in others between the IUGR cases and the control groups. Among these, there was a significant increase in fibrin deposition in intervillous spaces and around villi in idiopathic IUGR placentas. Although fibrinoid depositions were present in the control group placentas, they were observed more frequently in idiopathic IUGR placentas, consistent with previous research [7,13-19]. Bane and Gillan showed that massive perivillous fibrinoid deposition on the maternal side of the placenta, extending to engulf chorionic villi, results in atrophy and sclerosis of the affected villi, indicating chronic placental insufficiency [14].

Perivillous fibrin deposition in the intervillous space may result from maternal blood thrombosis. The villi embedded in this fibrin are not infarcted but are incapable of participating in nutrient transfer. Fibrin deposition tends to develop in placentas with good maternal blood supply, with greater blood flow leading to increased disorder, stasis, and perivillous fibrin deposition [20]. Park et al. reported that increased perivillous fibrin deposition may be due to inadequate maternal intravascular volume enlargement during pregnancy, leading to a low flow state [21]. Fibrin deposition may act as a barrier between fetal and maternal circulation, reducing the transfer of essential nutrients to the fetus and consequently causing IUGR [14,19].

In line with other studies, syncytial knots were present in both idiopathic IUGR and control placentas, but their number was significantly higher in the idiopathic IUGR group [15,21-23]. Syncytial knots are markers of compromised fetal circulation, resulting from reduced blood perfusion through the villi and an imbalance between the proliferation and apoptosis of syncytiotrophoblasts [20]. Elevated syncytial knots are associated with oxidative stress, including hypoxia and elevated reactive oxygen species production [24]. Villous agglutination in IUGR placentas may indicate reduced intervillous perfusion and early-stage infarction [25].

The present study found a significant increase in stromal fibrosis of villi in idiopathic IUGR placentas compared to control placentas, consistent with the findings of Almasry et al. [6] and others [7,13]. However, this contrasts with the results of Park et al. [21]. These differences may be due to variations in sample types, as the current study focused exclusively on idiopathic IUGR cases.

The current study showed a significant decrease in the mean number of terminal villi in idiopathic IUGR cases compared to controls. This is aligned with previous studies [6,13,26]. Other researchers did not find significant differences in the volume proportions of chorionic villous tissue between IUGR and normal pregnancies [27].

The study observed an insignificant increase in calcification in idiopathic IUGR placentas, consistent with previous research [13,21,22]. This contrasts with Nigam et al., who reported a significant increase in calcification in IUGR placentas [18]. The discrepancy may be due to Nigam et al.'s focus on normotensive IUGR cases, whereas the current study examined idiopathic IUGR. Calcification was observed in both control and IUGR groups, following a similar pattern in each. Since calcification is commonly seen in mature placentas and indicates placental aging, no clear link between IUGR and calcification was found [18]. Additionally, levels of chorangiosis in IUGR placentas were comparable to those in the control group. This is consistent with the findings of other studies [13,28].

The current study revealed insignificant differences in placentas of both idiopathic IUGR and control groups regarding chorangiosis, congestion of fetal capillaries, and fatty degeneration. İskender-Mazman et al. reported that chorangiosis and other pathological findings were rarely seen in placentas of IUGR [28]. Similarly, Park et al. and Yoo et al. found non-significant differences between case and control groups regarding chorangiosis [21,29]. Considering the fetomaternal results, the current study did not reveal significant differences in placental histological changes between the maternal age groups of the control and idiopathic IUGR cases. This is in line with the results of other researchers [6,16,26].

These histopathological changes in IUGR placentas may impair their ability to meet fetal needs, leading to fetal growth restriction. However, the fetus often compensates for placental dysfunction by reallocating nutrients to protect essential organs like the brain. As a result, head circumference is less impacted, suggesting that the placenta and fetus prioritize nutrients for brain development. The diverse microscopic changes seen in idiopathic IUGR placentas in the current study suggest reduced blood flow to the placenta and a smaller surface area for fetomaternal exchange. These conditions likely contribute to decreased oxygen and nutrient transfer, which can cause chronic placental insufficiency and restricted fetal growth and development [13,23].

Additional studies of idiopathic IUGR placentas are necessary, especially concerning the demographic, anatomic, immunologic, and molecular biological changes that could affect fetal and maternal exchange. Thus the investigation of the etiologies of idiopathic fetal growth restriction is important so that future care can be targeted at treatment and prevention.

## Conclusions

It is concluded from this study that the placenta in idiopathic IUGR pregnancies faced severe histopathological changes. The number of terminal villi was severely decreased as well as hyalinization. This was associated with an abnormal increase in syncytial knots, fibrinoid necrosis, and stromal fibrosis. There was also a slight increase in calcification, medial coat proliferation of medium-sized blood vessels, chorangiosis, and congestion of fetal blood capillaries. These various abnormal microscopic changes observed in the placenta in idiopathic IUGR lead to a decreased surface area for fetomaternal exchange, which may lead to chronic placental insufficiency. As a result, fetal growth and development are restricted. Therefore, it is necessary to plan more scientific and clinical research for IUGR babies.
